# National Health Insurance

**Published:** 1923-11

**Authors:** 


### NATIONAL HEALTH INSURANCE.

### A MOMENTOUS DECISION.

THE *Times* did not use exaggerated language when, in the course of a leading article, following the Minister's announcement of his offer of the new terms to the panel doctors, it said that " the decision which the doctors must now take is one of the most important in the history of their profession in this country." The Minister has let it be inferred from his communications that he will not allow the question of panel pay to go to arbitration. The Panel Committees, in conference, irked by this refusal and by the unhappy intervention of the Approved Societies, and also doubtless moved by genuine dissatisfaction at the terms, have decided to advise their constituents to send in their resignations. The Minister's offer of 8s. for five years, or 8s. 6d. for three years, is thus rejected. It now remains to be seen whether the individual doctors will respond in sufficient numbers to make this decision of the conference effective. It is a difficult situation for the individual doctor, but he may reflect that if his resignation is not used with success in the further bargaining which is foreshadowed, it could afterwards be withdrawn. It would be unfortunate if the Minister should find himself able to concede to threats what he was not able to agree to on merits.

### Why Not Arbitrate ?

We feel that the Minister's refusal or implied refusal to allow arbitration is unfortunate, however well founded, technically, his objections may be. We do not see why he should lay himself open to the charge that his terms do not bear close examination. They do ; and they seem to us to come out of an examination very well. In the first place, the Minister, finding himself assailed by the doctors for offering too little, and more fiercely by the Societies for offering too much, will reflect that his decision represents a happy mean. Again, it is not an arbitrary settlement which the Minister seeks to impose. In the long and closely-reasoned letter of October 3rd to the Insurance Acts Committee of the British Medical Association, there is an admixture of arithmetic and common sense which will satisfy public opinion. The doctors are fairly reminded in this document of two essential considerations which they are apt to overlook. The arbitration of 1920 took place " at a period of optimism and inflation," and we have all, for a long time since that date, lived in a different economic atmosphere. And the second consideration is this : that in the public service, especially in the administrative section, increases of pay since the war have not been designed to place the officials in the same economic position as before the war, but merely represent the extent to which " the financial exigencies of the country permitted of the effects of the rise in prices being mitigated in the case of the higher ranks of the public service."

### What the Minister's Offer Means.

A gross income of £1,800 a year in 1920 on the Civil Service analogy represented a pre-war income of £1,200, or thereabouts, and the equivalent at the present time is about £1,400. The amount of £1,800 was named by the Insurance Acts Committee in 1920 as offering a fair reward at that time for the whole time of a general practitioner. Let us see what the Minister offers. He is satisfied that a list of 2,000 persons, well and ill, involves on an average half a day's work for the doctor. If that be so, 2,000 persons represent half a year's income. A capitation fee of 8s. for that number produces £800 and a fee of 8s. 6d. produces £850. Thus, the Minister is offering the doctors an income for panel practice at the rate of £1,600 a year gross, for a five year's contract, or £1,700 a year for a three years' contract for the time given to their insurance work. We do not think that the Minister can concede any better terms. He may safely rely on public support in refusing to pay more than a rate of £1,700 a year gross, representing, after deduction of practice expenses, about £1,400 a year net.

### The Doctors' Reply.

The doctors, in their long reply to the Minister's letter, rather brushed aside this method of calculating the effective rate of payment, considered in the light of a yearly rate, although it was accepted and used by them in the 1920 arbitration ; their new method of determining the relative value of panel and private earnings on the basis of relative numbers of the population is ingenious but unconvincing. They also devoted much argument to a proposition which the Minister had nowhere put forward—that a doctor's duties were comparable to those of a higher civil servant. The Minister's argument, as we understand it, was simply that a doctor, engaged in a public service, might fairly expect his income to undergo a similar adjustment, in the changing economic conditions, to that of the income of a public servant.

### Looking After 2,000 Persons.

The new calculations on which it is estimated that 2,000 persons represent half a day's work throughout the year once again put in its proper perspective the number of persons for whom a doctor may assume responsibility. Records of work covering 736,000 insured persons have been examined, and it is found that a list of 2,000 persons involves, on average, about sixteen surgery attendances (including those for trifling ailments or even for certificates of renewal) and eight visits per working day. This calculation and the inferences to be drawn from it are of the essence of the question of the doctors' pay. If it is faulty, it must be seriously so to affect the broad result of the yearly rate offered to the doctors ; if it is approximately right, it is not only difficult to conceive of a wholesale refusal of service to be paid at such a rate, but it affords an interesting criterion of the way in which a salaried service could be financed if the necessity arose.

### How a Salaried Service Could be Financed.

If we take a town with 100,000 insured persons, it will be seen that 8s. 6d. a head produces £42,500. Such a sum would enable the equivalent of twenty-five whole-time doctors to be employed on a scale of salary ranging from £1,000 to £1,400 a year, or a mean rate of £1,200 a year, and would absorb £30,000 only, leaving £12,500 a year available for the expenses of central surgeries and for extra services. It is impossible to suppose that a sufficient number of doctors would not be attracted to such a service in a town area if the panel system should break down owing to the action of the doctors. In the rural districts the position is admittedly more difficult, and complete suspension of the service might be unavoidable in some places if the promised extra payments are not sufficiently attractive.

### The Societies Would not Succeed.

If we visualise a wholesale refusal of the doctors to accept the Minister's terms, it is idle to think of the Societies running a medical service for the whole country. They, more than anyone, have talked far and wide about the impossibility of doctors giving adequate attention to so many as 3,000 persons, and though the Minister's calculations show them to be wrong, they would find it difficult to put forward proposals on the basis of less than one doctor for 2,000 insured persons, as most of the Societies would want to run concurrently, a scheme for dependants. There are 14,000,000 insured persons. Where are the 7,000 doctors to come from? Indeed having regard to considerations of geography in the rural and semi-rural districts, and the need for more doctors, where are the 10,000 doctors to come from? And where is the territorial organisation of the thousands of Societies and branches?

### The Unhelpful Societies.

The intervention of the Approved Societies in this question has embittered the whole controversy and has been singularly unhelpful. What are we to think when the Consultative Council to the Minister, representative of every type of Approved Society experience, recommends an 8s. settlement, and the gathering of Approved Society representatives outside then declares for not a penny more than 7s. 3d.? Or when the Secretary of the General Federation of Trade Unions, in the columns of the *Times*, speaks disparagingly of the medical service and deprecates a payment of more than 7s. 3d., while at the most critical moment in the controversy the Labour Party memorandum comes to light which in effect supports a 9s. 6d. settlement? We feel that the doctors may at least reply on the support of public opinion in their resentment against the unhelpful, if not actually hostile attitude of those who claim to speak for the Approved Societies and against the growing aggressiveness of so many of them in their reiterated references to the funds contributed by employers, taxpayers and workers as "their funds." As to the attitude of the Societies at the meeting of October 17, when they indicated that if more than 7s. 3d. was to be found, the Minister would have to get it elsewhere—in other words, take it from the pockets of the taxpayer—we should say that no Minister would be found with the audacity to make such a proposal at the present time.

### The Alternatives to the Panel System.

The Minister may suspend the whole medical insurance service and hand over the available money to the insured persons or their representatives, the Approved Societies. But the Societies will not be able to provide a medical service if the Minister cannot do so. From the point of view of self-interest alone, it is obvious that doctors generally are not going to refuse 8s. 6d. offered by the Minister and accept the same amount (or less) from the Societies, some of whom affect to think that it is scandalous to offer more than 7s. 3d. But not only is self-interest in question; probably a large majority of the profession would agree with the *Times* when it says that there have always been weighty objections to contract practice, and that if it is to degenerate into the control of a learned profession by a group of Benefit Societies, those objections would be enormously enhanced. A medical service organised by the Approved Societies is not therefore a practical alternative to the panel system. It would break down, and the whole National Health Insurance scheme would be discredited. Other alternatives are a salaried service organised by the Minister direct or through Insurance Committees—and we have seen above that, in town areas, there would be ample funds—and a service organised by the doctors themselves. If we look first at the latter proposition, it seems obvious that Parliament is not going to provide extra funds in order, as some would not hesitate to express it, to finance a strike; 7s. 3d. is the utmost that would be available. And as the insured persons can only deal in the mass through their Societies, we seem to be thrown back more or less on what we have already declared would break down.

### A Stop-Gap Salaried Service.

We do not think that a State salaried service for the country generally is at the present time a practical proposition. Salaried services here and there, arranged by the Minister through the Insurance Committees, are practical and probable where there is a partial suspension of the panel system. And it is as well to remember that the Minister has power to suspend the panel system for any area or any part of an area, however small, and is entirely unfettered as to the way in which the gap shall be stopped. It would be correct to describe any such patchy arrangements as mere stop-gaps; and if they were to be at all numerous, it would really be better to have a complete upheaval.

### An Overhaul and a New Atmosphere.

A complete suspension of medical benefit while the promised Royal Commission overhauls the machinery would in fact not be an unmixed evil. That it would be bad for the insured person's pockets would be clear, but the doctors would not allow their bodily ills to go unattended; that it would be bad for the doctors' peace of mind, we are satisfied. But if it paved the way for a new medical service, organised as part of the local health services of the country, in a new atmosphere, after a chastening process all round, it would be well worth while.

